# The roles and mechanisms of coding and noncoding RNA variations in cancer

**DOI:** 10.1038/s12276-024-01307-x

**Published:** 2024-09-02

**Authors:** Sang Yean Kim, Min Jeong Na, Sungpil Yoon, Eunbi Shin, Jin Woong Ha, Soyoung Jeon, Suk Woo Nam

**Affiliations:** 1 Department of Pathology, College of Medicine, The Catholic University of Korea, Seoul, Republic of Korea; 2 Functional RNomics Research Center, The Catholic University of Korea, Seoul, Republic of Korea; 3 NEORNAT Inc., Seoul, Republic of Korea; 4 Department of Biomedicine & Health Sciences, Graduate School, The Catholic University of Korea, Seoul, Korea

**Keywords:** Cancer epigenetics, Gene regulation

## Abstract

Functional variations in coding and noncoding RNAs are crucial in tumorigenesis, with cancer-specific alterations often resulting from chemical modifications and posttranscriptional processes mediated by enzymes. These RNA variations have been linked to tumor cell proliferation, growth, metastasis, and drug resistance and are valuable for identifying diagnostic or prognostic cancer biomarkers. The diversity of posttranscriptional RNA modifications, such as splicing, polyadenylation, methylation, and editing, is particularly significant due to their prevalence and impact on cancer progression. Additionally, other modifications, including RNA acetylation, circularization, miRNA isomerization, and pseudouridination, are recognized as key contributors to cancer development. Understanding the mechanisms underlying these RNA modifications in cancer can enhance our knowledge of cancer biology and facilitate the development of innovative therapeutic strategies. Targeting these RNA modifications and their regulatory enzymes may pave the way for novel RNA-based therapies, enabling tailored interventions for specific cancer subtypes. This review provides a comprehensive overview of the roles and mechanisms of various coding and noncoding RNA modifications in cancer progression and highlights recent advancements in RNA-based therapeutic applications.

## RNA Modifications in Cancer: Impact on Tumor Progression and Therapy

In the intricate field of cancer study, researchers investigate how changes in RNA, a molecule that carries genetic instructions, contribute to cancer. Researchers explores these RNA changes, their mechanisms, and their role in cancer development. The authors review various ways RNA can be modified, including methylation, editing, and alternative splicing. The authors underline how these RNA changes can impact cancer development by affecting gene activity, protein creation, and cell behavior. The review’s key findings suggest that RNA changes are deeply involved in cancer’s start, development, and treatment response. By charting the landscape of RNA changes in cancer cells, the authors aim to pave new paths for diagnosis, prognosis, and treatment. They conclude targeting RNA changes offers a promising strategy for developing innovative cancer treatments, marking a significant progress in our approach to fighting this disease. This summary was initially drafted using artificial intelligence, then revised and fact-checked by the author.

## Introduction

RNA variations are categorized into alternative splicing, RNA editing, microRNA variation, RNA methylation and alternative polyadenylation, all of which yield alternative transcripts^1^. These variations, characterized by various enzymatic processes that act on pre-RNA molecules, generate a plethora of mature RNA forms with diverse functional implications. This diversity in posttranscriptional RNA modifications underlies a wide array of individual or pathological phenotypes, including organ specificity and developmental stages^2,3^. Significant milestones in RNA variation research include the discovery of alternative splicing (AS) transcripts from premessenger RNA (pre-mRNA) in 1977 and microRNAs (miRNAs) in 1993^4^. Coding RNAs (messenger RNAs, mRNAs) serve as templates for protein synthesis via ribosomes. Therefore, variations in pre-mRNAs can profoundly impact protein function, influencing mRNA stability and subsequent protein production within cells. These variations can also disrupt miRNA-mediated translational inhibition, mRNA‒protein interactions, and the subcellular localization of mRNAs or their protein products.

Noncoding RNAs (ncRNAs) encompass a diverse group of functional RNA molecules that are not translated into proteins. ncRNAs include structural RNAs such as ribosomal RNA (rRNA) and transfer RNA (tRNA), as well as regulatory RNAs such as miRNAs, small interfering RNAs (siRNAs), Piwi-interacting RNAs (piRNAs), enhancer RNAs (eRNAs), long noncoding RNAs (lncRNAs), circular RNAs (circRNAs), and vault RNAs (vRNAs). Enzyme-mediated RNA modifications, including methylation, alternative splicing, RNA editing, and alternative polyadenylation (APA), generate various isoforms of mRNAs or ncRNAs^5^. Other RNA modifications include acetylation, pseudouridine modification, and circularization^6,7^.

**Fig. 1:**
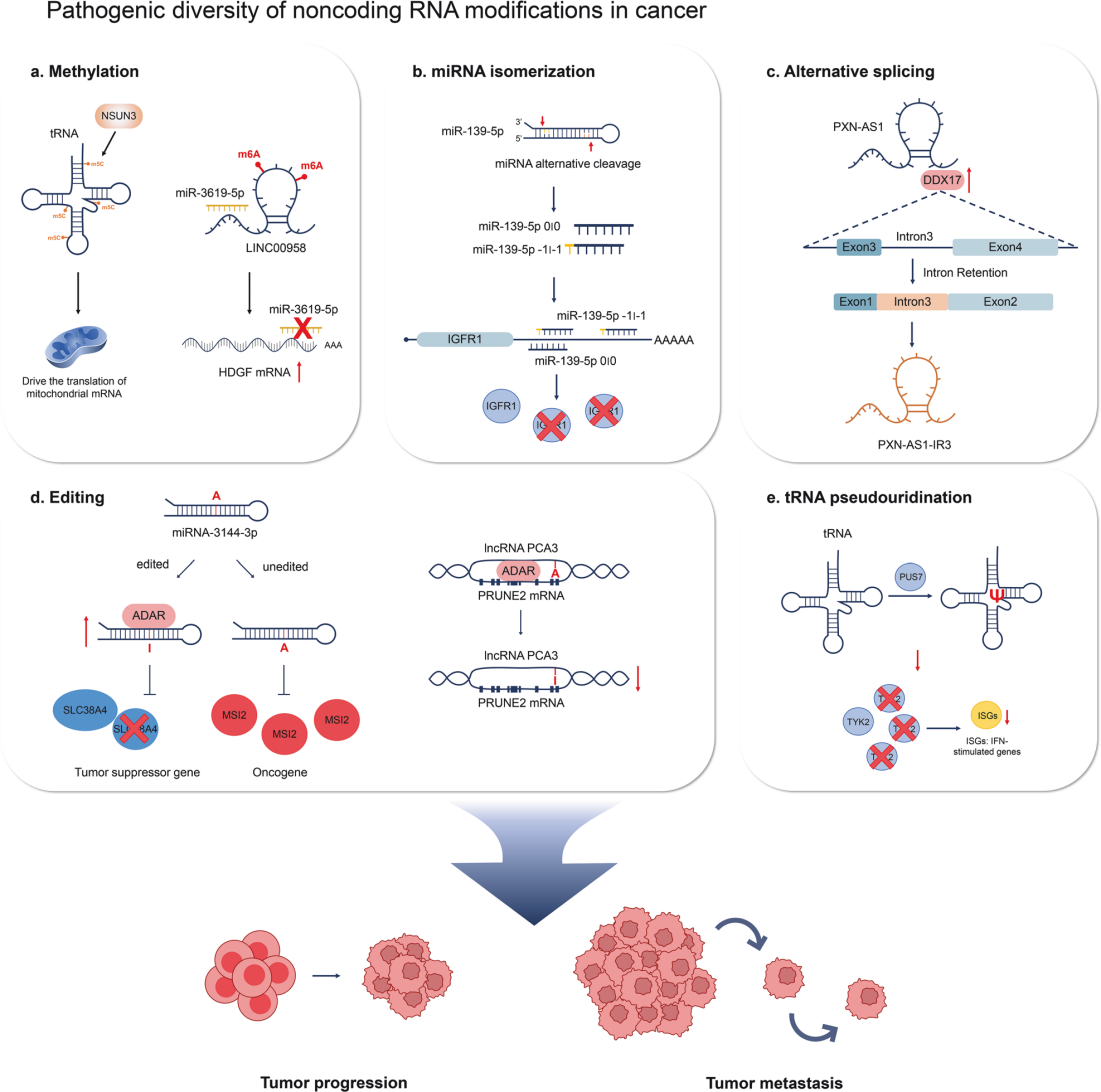
Roles and mechanisms of representative noncoding RNA modifications in tumorigenesis. The functional diversity of noncoding RNAs in cancers results from **a** methylation, **b** miRNA isomerization, **c** alternative splicing, **d** editing, or **e** tRNA pseudouridination. These modifications play crucial roles in tumorigenesis, particularly in specific cancer subtypes, influencing the pathogenesis and progression of tumors.

Recent investigations have identified 5′ and 3′ isomerizations of miRNAs resulting from various pre-RNA digestion processes, with aberrant expression increasingly implicated in disease phenotypes (Fig. 1 and Table 1). These diverse RNA variations have been associated with various human diseases, including obesity, diabetes, Alzheimer’s disease, systemic lupus erythematosus (SLE), hypertension, and cancer^8,9^. Dysregulation of specific RNA modifications is particularly notable in cancer, where they play pivotal functional roles in tumorigenesis, tumor invasion, metastasis, angiogenesis, hematological malignancies, and the establishment of tumor microenvironments conducive to hypoxia or immune evasion^10–12^. Moreover, specific RNA modifications have been associated with drug resistance and serve as clinically relevant diagnostic or prognostic biomarkers^13,14^.

**Table 1:** Categories of coding and noncoding RNA modifications in cancers.

Category		Event	Modification	Ref.
coding RNA	mRNA	alternative polyadenylation (APA)		^25,26,28,32^
		alternative splicing		^36,37,39^
		editing	A-to-I	^43,45–47^
			C-to-U	^50,51^
		methylation	N6-methyladenosine (m6A)	^53–56^
			N7-methylguanosine (m7G)	^57^
		N4-acetylcysteine (ac4C)		^61–63^
small noncoding RNA	tRNA	methylation	N1-methyladenosine (m1A)	^80^
			5-methylcytideine (m5C)	^81^
		pseudouridination		^82^
	miRNA	methylation	N6-methyladenosine (m6A)	^71,90,91^
			5-methylcytideine (m5C)	^92^
			N7-methylguanosine (m7G)	^93^
		editing	A-to-I	^47,87,88^
		isomiRNA		^95–102^
large noncoding RNA	lncRNA	alternative splicing		^112–114^
		editing	A-to-I	^115,118–120^
		methylation	N6-methyladenosine (m6A)	^121–123,126,127^
			5-methylcytideine (m5C)	^128,129^
	circRNA	methylation	N6-methyladenosine (m6A)	^134^
			5-methylcytideine (m5C)	^137,138^
		editing		^146^

Therapeutic strategies targeting aberrant RNA modifications, including siRNAs, miRNAs, inhibitory antisense oligonucleotides (ASOs), plasmid DNA, mRNA, splicing-modulatory ASOs, and clustered regularly interspaced short palindromic repeats/CRISPR associated gene (CRISPR/Cas) systems, have garnered significant interest^15–17^. Notably, the FDA approval of ONPATTRO™ (patisiran) in 2018 marked a milestone in siRNA therapeutic development, opening new avenues for siRNA-based cancer therapies^18^. Numerous therapeutic programs targeting various oncogenes and pathways, including KRAS mutants, VEGF, MYC oncogene, and PLK1, are underway^19–21^. This review aims to comprehensively elucidate the roles and mechanisms of various RNA modifications in cancer progression and explore their functional implications and therapeutic potential. Understanding the intricate mechanisms of RNA modifications in specific cancer subtypes could enhance our understanding of cancer biology and facilitate the development of innovative therapeutic strategies.

## Coding RNA modifications

### Variations in mRNA polyadenylation

The modification of the 3′ end of mRNA represents a pivotal stage in the maturation process of pre-mRNA. This process, encompassing cleavage and polyadenylation (CPA), constitutes the final step in refining the pre-mRNA 3’ end. Alternative polyadenylation (APA) occurring within the 3′ untranslated region (3’UTR) introduces variations in mRNA length, resulting in transcripts of various lengths^22,23^. Notably, more than 70% of human genes possess three or more APA sites, allowing for cleavage at different sites and the production of mRNA transcripts of diverse lengths^24^. This diversity in mRNA length correlates with increased stability and higher expression levels compared to those of other noncoding RNA transcript types. The length of the 3′UTR significantly influences mRNA localization, transport, stability, translation, nuclear export, cytoplasmic localization, interactions with miRNAs, and translation efficiency^23,25–27^. Dysregulation of APA has been associated with several human diseases, with cancer exhibiting the highest incidence, followed by neurological, immunological, and musculoskeletal disorders^28^. Of particular interest is the ability of APA to modulate cyclin D1 levels, with patients expressing isoforms of cyclin D1a mRNA featuring short 3′UTRs exhibiting a shorter median survival^29^. Additionally, the truncated proteins resulting from intronic polyadenylation often lack the tumor-suppressive functions of their full-length counterparts (such as DICER and FOXN3) and, in some cases, exhibit oncogenic properties (such as CARD11, MGA, and CHST11)^25^.

**Fig. 2:**
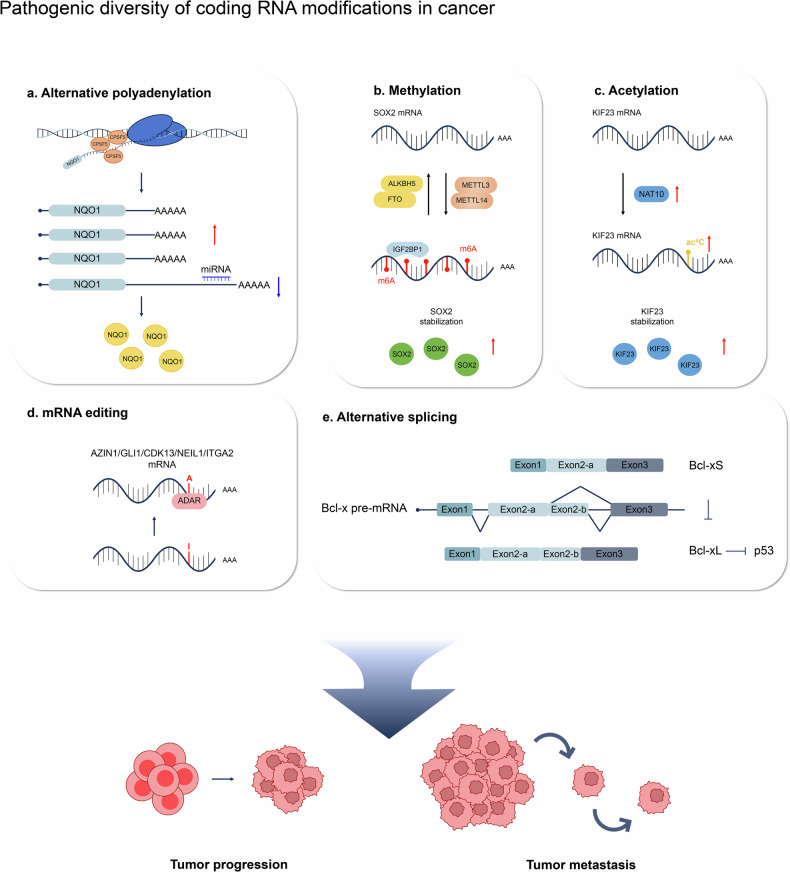
Roles and mechanisms of representative coding RNA modifications in tumorigenesis. Functional diversity of mRNAs in cancers resulting from **a** alternative polyadenylation, **b** methylation, **c** acetylation, **d** mRNA editing, or **e** alternative splicing. These modifications play crucial roles in tumorigenesis, particularly in specific cancer subtypes, influencing the pathogenesis and progression of tumors.

A protein complex comprising cleavage and polyadenylation specificity factors (CPSFs) recognizes the polyadenylation signal sequence (PAS). This complex, consisting of four large subcomplexes—CPSF, CSTF, CFIm, and CFIIm—comprising a total of 20 proteins, recruits other protein complexes, including CSTF, to the polyadenylation signal sequence. The poly(A) tail, ranging from approximately 100 to several hundred nucleotides in length, plays a crucial role in mRNA stability and regulation^24,30,31^. Given the involvement of APA regulatory factors in cancer, mutations in these factors have been identified in various cancers. For instance, knocking down CPSF5 resulted in the shortening of more than 1450 mRNA 3′UTRs in glioblastoma cells, thereby increasing tumorigenesis^32^. Notably, CPSF6-induced tumorigenic activity is mediated by specific mRNA isoforms featuring short 3′UTRs in hepatocellular carcinoma (HCC)^26^. Investigations into APA regulation in cancer are promising for elucidating disease mechanisms, identifying potential diagnostic markers, and guiding the development of targeted therapies (Fig. 2 and Table 1).

### Variations in mRNA splicing

Pre-mRNAs, which are composed of exons and introns, undergo AS, resulting in the generation of various mature mRNA forms^33,34^. AS involves the removal or combination of exons within pre-mRNAs, leading to different protein isoforms with distinct functional roles. Although AS typically contributes to developmental or tissue-specific features of alternative protein isoforms, dysregulation of AS can disrupt critical cellular pathways involved in cell cycle regulation, apoptosis, DNA repair, and environmental adaptations, thereby contributing to tumorigenesis. This phenomenon has been observed across various cancer types originating from different organs^34,35^. Notably, there is a greater incidence of AS in cancer tissues than in normal tissues. For instance, an extensive analysis of 8000 tumor samples across 32 cancer types revealed a 30% increase in AS in cancer tissues^36^. The expression levels of splicing isoforms of BCL-xL, which play roles as antiapoptotic factors, are upregulated in tumor cells^37^.

The splicing of pre-mRNAs is orchestrated by the spliceosome, which comprises five small nuclear ribonucleoproteins (snRNPs): U1, U2, U4, U5, and U6^38^. Each snRNP consists of small nuclear RNAs (snRNAs) and several associated proteins. Dysregulation of splicing factors (SFs), either through overexpression or mutation, is correlated with aberrant AS in cancer. For instance, the overexpression of SFs such as SRSF1, hnRNP A1, and SRSF3 has been reported in breast cancer and lung cancer^39^. These alterations can result in the generation of oncogenic splice variants that promote tumorigenesis. Conversely, mutations in SFs such as SF3B1, U2AF1, and SRSF2, which are frequently observed in myelodysplastic syndromes and chronic lymphocytic leukemia, can disrupt normal splicing processes, leading to the production of abnormal proteins that drive cancer progression. In addition, SFs regulate AS by binding to specific RNA sequences or structures near splice sites, influencing the recruitment and assembly of the spliceosome. For example, SR proteins typically bind to exonic splicing enhancers (ESEs) and promote the inclusion of exons by facilitating spliceosome assembly at adjacent splice sites^40^. hnRNP proteins, conversely, often bind to exonic or intronic splicing silencers (ESSs or ISSs, respectively) and inhibit splice site usage, leading to exon skipping^41^. These interactions are crucial for the precise regulation of AS and can be perturbed in cancer, resulting in the expression of splice variants that contribute to oncogenic processes such as increased cell proliferation, resistance to apoptosis, and enhanced metastasis. Moreover, emerging evidence suggests that aberrant AS can functionally interact with other genetic and epigenetic changes to drive tumorigenesis (Fig. 2 and Table 1).

### Editing variations in mRNA

Adenosine-to-inosine (A-to-I) editing in mRNA entails a site-specific nucleotide substitution in pre-mRNA before splicing^42^. The functional outcomes of mRNA editing include alterations in amino acid sequences if editing occurs in exons. Editing within intronic regions enhances mRNA stability, leading to increased production of protein products. Additionally, editing in intronic areas can alter AS events, resulting in the production of different isoforms. Editing also regulates mRNA stability by disrupting miRNA binding in the 3′UTR of mRNA or recruiting other proteins to Alu-rich regions in the 3′UTR^43^.

RNA editing enzymes exhibit tissue-specific expression patterns, underscoring the pivotal role of RNA editing in proper tissue development and determination. ADAR1 and ADAR2 are key enzymes involved in the A-to-I editing process. Aberrant expression patterns of ADAR1 or ADAR2 editing enzymes are strongly associated with cancer development. ADAR1 can undergo SUMOylation as an upstream regulatory mechanism, thereby repressing its A-to-I RNA editing activity^1^. For instance, ADAR1 colocalizes with SUMO-1 and is modified by SMO-1 at lysine 418, leading to reduced enzyme editing activity in vitro^43^.

ADAR1 is frequently overexpressed in breast, lung, liver, and esophageal cancers and chronic myelogenous leukemia and is positively correlated with cancer progression and malignant phenotypes, such as invasion^44^. ADAR1 edits the mRNAs of AZIN1, BLCAP, NEIL1, GLI1, ITGA2, and CDK13, promoting cancer progression, while editing of the GABRA3 and CCNI genes by ADAR1 can suppress tumorigenesis^45,46^. Another crucial editing enzyme, ADAR2, has been shown to downregulate glioblastoma and liver cancer. Increased malignancy and poor prognosis in gastric cancer are associated with ADAR2 downregulation^47^.

Enzyme-mediated cytidine-to-uridine (C-to-U) editing is another form of mRNA editing. APOBEC1 and APOBEC3 are representative enzymes among the 11 known members and are capable of binding to single-stranded RNA (ssRNA)^48,49^. APOBEC enzymes involved in C-to-U editing are implicated in tumorigenesis. For instance, APOBEC1 overexpression in the livers of transgenic animals induces HCC^50^. Mutations in RBM47, an APOBEC1 cofactor, have been linked to breast cancer progression^51^ (Fig. 2 and Table 1).

### Methylation variations in mRNA

Over 170 RNA modifications have been identified, two-thirds of which are reversible RNA methylations. RNA methylation occurs at various locations within RNA molecules, including the RNA cap, 5′UTR, coding region, and 3′UTR. Representative RNA methylations include N6-methyladenosine (m6A), N7-methylguanosine (m7G), 5-methylcytidine (m5C), N1-methyladenosine (m1A), and 2′-O-methylation (2′-O-Me)^52^. These modifications contribute to mRNA stability, a crucial factor in tightly regulating gene expression.

Of particular importance, m6A is the most abundant internal mRNA modification, with consensus sequences identified as Gm6AC or Am6AC. m6A modification serves a vital biological function in regulating mRNA metabolism, including processes such as structure, folding, stability, maturation, splicing, translocation, nuclear export, degradation, and translation. Alterations in m6A modification can induce changes in RNA secondary structures and play critical roles in various pathological and biological processes, notably tumorigenesis and progression^53^. Proteins involved in m6A modification, including writers, readers, and erasers, have been shown to play pivotal roles in tumorigenesis, as these modifications are reversible processes. For instance, reports indicate that METTL3, a member of the writer family, and the IGF2BP family, which act as readers, are implicated in HCC progression through aberrant m6A modifications of RNAs^54,55^. In HCC, METTL14 interacts with DGCR8 to suppress metastasis by promoting miRNA-126 processing in an m6A-dependent manner. Conversely, FTO, an m6A demethylase, reduces the mRNA stability of APOE by decreasing its m6A modification, thereby inhibiting glycolysis and growth in papillary thyroid carcinoma. Similarly, the m6A demethylase ALKBH5 reduces the mRNA stability of PKMYT1 by decreasing its m6A modification, thereby inhibiting the invasion of gastric cancer cells. SUMOylation can regulate the activities of the writer METTL3, the reader YTHDF2, and the eraser ALKBH5^56^.

m7G is present in various molecules, including the mRNA 5′ cap and interior. In HCC, m7G and the ubiquitination of p53 lead to decreased p53 expression and promote tumor progression. m7G-cap modification is associated with tumorigenesis. The cap methylation of some oncogenic mRNAs enhances their nuclear export and translation. For instance, RNMT enhances the translation of Cyclin D1 mRNA by promoting its cap methylation, ultimately promoting mammary epithelial cell transformation^57^ (Fig. 2 and Table 1).

### Other modification-derived variations in coding RNAs

The N4-acetylcysteine (ac4C) modification, catalyzed by NAT10, primarily occurs in the coding regions of mRNA, where it enhances stability and translation^52,58–60^. Variations in ac4C mRNA levels have been implicated in various human diseases, particularly cancer. For instance, the interaction of KIF23 mRNA with ac4C is positively correlated with the promotion of colorectal cancer cells^61^. Helicobacter-induced NAT10 stabilizes MDM2 mRNA via ac4C, contributing to the progression of gastric cancers^62^. Increased expression of NAT10 in colon cancer cells is correlated with shorter patient survival^63^ (Fig. 2 and Table 1).

Noncoding RNAs (ncRNAs), such as miRNAs, lncRNAs, and circRNAs have been reported to encode peptides that play functional roles in tumorigenesis^64^. For example, an endogenous peptide, LINC00998, can produce a 7.3 kDa peptide, SMIM30, on the cell membrane, exerting a positive effect during HCC progression^65^. Another example is the small regulatory peptide of STAT3 (ASRPS), encoded by LINC00980, which is downregulated in triple-negative breast cancer (TNBC) and is associated with a poor prognosis^66^. Additionally, a long noncoding RNA-encoded peptide, PINT87aa, which is induced by p53, plays a suppressive role in HCC^67^.

## Small noncoding RNA modification

Representative structural small noncoding RNAs include RNA polymerase III-derived tRNA and 5 S rRNA. tRNA is among the most conserved and abundant RNA species and undergoes posttranscriptional modifications by tRNA-modifying enzymes^68,69^. Methylated 5 S rRNA has been identified in plants but not in cancer cells. However, 5 S rRNA overexpression is associated with various cancer types^70–72^, suggesting that cancer-related variations in 5 S rRNA may be identified in the future.

**Fig. 3:**
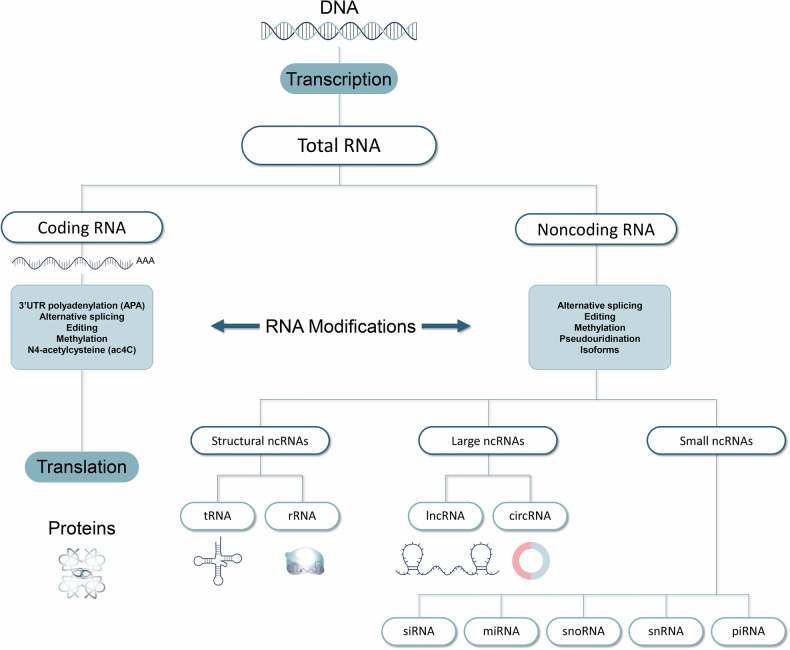
Posttranscriptional RNA diversity according to modified coding or noncoding RNA variations. Various isoforms of mRNAs or ncRNAs can be generated from enzyme-mediated RNA modifications such as methylation, alternative splicing, RNA editing, and alternative polyadenylation. These different types of RNA variations have also been demonstrated to be connected with tumorigenesis. tRNA, transfer RNA; rRNA, ribosomal RNA; lncRNA, long noncoding RNA; circRNA, circular RNA; siRNA, small interfering RNA; miRNA, microRNA; snoRNA, small nucleolar RNA; snRNA, small nuclear RNA; piRNA, piwi-interacting RNA.

RNA polymerase II-derived miRNAs and siRNAs are two major classes of regulatory small noncoding RNAs. miRNAs are abundant in many mammalian cell types and resemble siRNAs of the RNA interference (RNAi) pathway. miRNAs originate from regions of RNA transcripts that fold back on themselves to form short hairpins, whereas siRNAs are derived from longer regions of double-stranded RNA. Despite their similarities, miRNAs typically silence genes by repressing translation and have broader specificity, while siRNAs usually exhibit greater specificity by cleaving mRNA before translation with 100% complementarity^73^. Dysregulation of miRNA modifications or isomerizations has been implicated in various types of tumors^74^. Additionally, piRNAs and vRNAs are small noncoding RNA species involved in the regulation of transposon silencing and drug resistance. Y RNA, another small noncoding RNA, acts as an initiation factor for DNA replication and is overexpressed in some tumors. Recently, small ncRNAs have been shown to undergo glycosylation on membranes, resulting in the formation of glycoRNAs, which are important for the immune system^75,76^ (Fig. 3).

### tRNA modifications and their variations

tRNAs usually undergo methylation^75,77^ and pseudouridination^78^. These modifications are highly conserved and play critical roles in tRNA structure, ribosome binding affinity, decoding activity, and the maintenance of anticodon folding and pairing functions^68,79^. These modifications are essential in tumorigenesis. For instance, m1A modifications in tRNAs have been identified as significant factors for T-cell proliferation^80^. Additionally, the methyltransferase NSUN3, which catalyzes m5C tRNA modifications, is a predictor of lymph node metastasis and poor patient outcomes in head and neck cancer^81^ (Fig. 1 and Table 1). The knockdown of PUS7 induces a reduction in the pseudouridynation of tRNA, consequently upregulating the expression of proteins within the TYK2 pathway. This, in turn, hampers cell growth and fosters antitumorigenic effects in glioblastoma^82^.

tRNA-derived small RNAs (tsRNAs) represent a growing category of small noncoding RNAs generated from the cleavage of mature tRNAs or tRNA precursors by the enzyme ELAC2. These tsRNAs are classified as tRFs based on whether cleavage occurs at the 3′ or 5′ end^83^. tsRNAs exhibit both oncogenic and tumor suppressor functions and play vital roles in the development and progression of various cancers. Increasing evidence suggests that extracellular tsRNAs have potential as diagnostic and prognostic biomarkers for cancer through liquid biopsy^84,85^.

### miRNA modifications and their variations

miRNAs are small transcripts, typically 18–22 base pairs long; and pre-miRNA processing is crucial for producing mature miRNAs. miRNA regulation requires perfect base pairing within the seed region (positions 2–8), meaning that a single nucleotide change can significantly alter miRNA target recognition^86^. In cancer, RNA editing and methylation of miRNAs can significantly impact their function. For example, ADAR2-catalyzed RNA editing occurs within the seed region of miR-379-5p. This site is underedited in tumors compared with normal tissues, with higher editing levels correlating with improved patient survival across cancer types^87^. In liver cancer, ADAR1-dependent editing of miR-3144-3p increases MSI2 expression and suppresses SLC38A4^88^. ADAR1 is overexpressed in HCC, leading to edited miR-3144-3p not targeting its original oncogene MSI2, thus allowing MSI2 overexpression in HCC^88^. Other miRNAs, such as let-7 and miR-376a, are also implicated in tumorigenesis through their editing^47^.

The distinct methylation patterns of mature miRNAs are notable in gastrointestinal cancers^89^. METTL3, for instance, methylates pri-miRNAs, and m6A methylation is crucial for efficient miRNA production in cancer cells^71,90,91^. m5C modification impairs the tumor-suppressor function of miRNA-181a-5p, which is correlated with poor prognosis in glioblastoma patients^92^. Following pre-miRNA processing, m7G methylation can persist on mature miRNAs, influencing their function. METTL1 promotes let-7 miRNA processing via m7G methylation^93^. Furthermore, TruB1, a predominant mammalian tRNA pseudouridine synthase, binds the stem loop of pri-let-7, enhancing its interaction with the miRNA processor protein DGCR8. This interaction promotes the maturation of let-7 miRNA family members, which are critical for inhibiting cell growth^94^.

### miRNA length variations with 5′ isomiRNA or 3′ isomiRNA isoforms

Multiple types of isomiRNAs, which exhibit variations in length due to the addition or loss of nucleotides at the 5′ end, 3′ end, or both, exist within cells^95,96^. These isomiRNAs can differ significantly from their canonical miRNA counterparts in terms of abundance, stability, and function. Some isomiRNAs are ubiquitously expressed, while others show tissue-specific expression and abundance. This variation suggests that isomiRNAs play roles in the pathogenesis and progression of various cancers and contribute to the molecular heterogeneity of cancer^95,97–99^. The dysregulation of isomiRNAs results in increased heterogeneity of miRNA isoforms with altered 5’ or 3’ ends, leading to the recognition of distinct target sites due to their shifted seed sequences. Studies using breast cancer datasets from The Cancer Genome Atlas (TCGA) revealed that compared with canonical miRNA patterns, isomiRNA expression patterns could better distinguish tumors from normal breast tissue. This distinct expression indicates the potential of isomiRNAs as more precise biomarkers for cancer diagnosis^97,100^. Functionally, isomiRNAs can be redundant or divergent from their canonical miRNAs. For instance, miR-139-5p variants have been reported to synergistically suppress tumor development and progression by targeting IGF1R in HCC^101^. In colon cancer, a significant number of differentially expressed isomiRNAs were identified compared to those in standard samples. Notably, approximately 50% of the detected miRNAs exhibited increased expression of their variants compared to the canonical forms. Overall, 2,451 isomiRNAs derived from 343 unique miRNAs were found to be differentially expressed in tumor versus standard samples, highlighting their substantial role in cancer biology^96,102^ (Fig. 1 and Table 1).

## Long noncoding RNA modification

Long noncoding RNAs (lncRNAs) are transcripts longer than 200 nucleotides that are generated by RNA polymerase II. Consequently, they undergo processes similar to those of mRNAs, including splicing, capping, polyadenylation, and editing^103,104^. Over 30,000 lncRNAs have been identified, two-thirds of which do not overlap with protein-coding transcripts; these are termed long intergenic noncoding RNAs (lincRNAs)^105,106^. LncRNAs have recently been recognized as central regulators of gene expression across various genes^107^. Aberrant expression or modulated activity of lncRNAs has been implicated in several cancer-related processes, including proliferation, growth suppression, motility, immortality, angiogenesis, and cell viability^108–110^. Moreover, specific lncRNA polymorphisms are associated with particular cancer types, highlighting their potential as biomarkers and therapeutic targets in oncology^111^ (Fig. 3).

### Variations in lncRNA splicing in cancer

AS of lncRNAs generates different isoforms that can have distinct functions in cancer^112,113^. For instance, the multiexon lncRNA PXN-AS1, regulated by the splicing factors MBNL3 and DDX17, produces multiple isoforms in HCC. MBNL3 promotes the inclusion of exon 4 in PXN-AS1, resulting in the formation of the PXN-AS1-L isoform, which inhibits myeloid cell leukemia (MCL)-mediated cell apoptosis in a PXN-dependent manner. In contrast, DDX17 induces the retention of intron 3 in PXN-AS1, creating the aberrant isoform PXN-AS1-IR3, which promotes HCC metastasis by inducing MYC transcription activation^114^. Recent studies have shown that some pre-mRNAs are bifunctional, serving as precursors for both mRNAs and lncRNAs. For example, PNUTS pre-mRNA encodes both PNUTS mRNA and lncRNA-PNUTS. In breast cancer, the production switches to lncRNA-PNUTS, which acts as a competitive sponge for miR-205, promoting epithelial–mesenchymal transition (EMT). In lung adenocarcinoma (LUAD), the bifunctional PD-L1 pre-mRNA produces PD-L1-lnc, a lncRNA isoform induced by IFNγ. PD-L1-lnc binds to MYC, enhancing its transcriptional activity, which activates downstream genes and promotes LUAD cell proliferation and invasion^114^ (Fig. 1 and Table 1).

### Variations in RNA editing in lncRNAs in cancer

The involvement of lncRNAs in oncogenes and tumor suppressors is significantly influenced by A-to-I RNA editing, with these modifications being markedly altered in cancer cells^115^. ADAR1, a key enzyme in this process, can modulate RNA expression levels by interacting with other RNA-binding proteins, such as Dicer and HuR^116,117^. Additionally, ADAR1 edits double-stranded RNA (dsRNA) within lncRNAs, altering their structure and subsequently affecting the binding of downstream target miRNAs.

Prostate cancer antigen 3 (PCA3), a long noncoding RNA, is notably upregulated in human prostate cancer^118^. PCA3 regulates PRUNE2 levels through a distinctive mechanism involving the formation of PRUNE2/ADAR1-edited PCA3 double-stranded RNA. ADAR1 editing at multiple sites within the PCA3/PRUNE2 duplex reduces PRUNE2 levels while increasing PCA3 expression^119^. This alteration promotes the proliferation, adhesion, and migration of cancer cells, underscoring the critical role of RNA editing in lncRNA-mediated cancer progression^44,118,120^.

### Methylation variation in lncRNAs in cancer

The interplay between lncRNAs and m6A modifications plays a crucial role in cancer^121–123^. These modifications impact lncRNA functions by altering their structure and accessibility to proteins, mediating gene transcriptional regulation, affecting mRNA precursor splicing, regulating lncRNA stability, and influencing lncRNA translation^124,125^. For instance, the lncRNA DIAPH1-AS1 promotes the growth and metastasis of nasopharyngeal carcinoma (NPC) in an m6A-dependent manner. Additionally, METTL14 suppresses the proliferation and metastasis of colorectal cancer cells by downregulating the oncogenic lncRNA XIST^126^. In HCC, m6A-mediated upregulation of LINC00958 enhances lipogenesis and serves as a potential nanotherapeutic target^127^. In addition, aberrant m5C modification of H19 lncRNA, mediated by NSUN2, is associated with poor differentiation in HCC^128^. m5C-methylated H19 lncRNA stimulates MYC and modulates the Ras signaling pathway and the cell cycle. Furthermore, six m7G-related lncRNAs have been identified as prognostic markers for HCC, providing valuable insights for immunotherapy and chemotherapy^129^ (Fig. 1 and Table 1).

### Other modifications in lncRNAs

Pseudouridine is the most abundant internal RNA modification in stable noncoding RNAs^130,131^. Several pseudouridine sites are present in human lncRNAs, including LRRC75A, LRRC75A-AS1, MALAT1, XIST, and SNHG1^132^. In non-small cell lung cancer (NSCLC), the lncRNA PCAT1 is significantly upregulated and interacts with DKC1, a pseudouridine synthase. This interaction influences cell proliferation, invasion, and apoptosis in NSCLC via the VEGF/AKT/Bcl-2/Caspase 9 pathways^133^.

### Circular RNA and variation in cancer

Circular RNAs (circRNAs) represent closed forms of single-stranded RNAs in which the 5′ and 3′ ends are joined by covalent bonds^134^. This circular structure confers stability to circRNAs by protecting them from exonucleases, thereby increasing RNA stability. While circRNAs can function as gene expression regulators in the nucleus, their most well-known roles are in the cytoplasm and include acting as miRNA sponges, protein sponges, protein scaffolds, and even templates for protein production^135^. Additionally, circRNAs can be packaged into exosomes and circulate throughout the body, serving as signaling molecules. Recent studies have identified altered circRNAs in the serum of patients with gastric cancer, indicating the potential of circRNAs in body fluids as reliable biomarkers^136^. Furthermore, posttranscriptionally methylated circRNAs, including m6A and m5C modifications, are closely linked to the tumorigenesis of HCC^134,137,138^.

These insights underscore the diverse functions and potential clinical applications of ncRNAs in cancer biology, highlighting their roles as both biomarkers and therapeutic targets.

## Regulatory interactions between RNAs and noncoding RNAs in cancer

### Regulatory interactions between lncRNAs and small noncoding RNAs in cancer

The concept of competing endogenous RNA (ceRNA) was first proposed in 2011 and suggested that ceRNAs can regulate the abundance of other RNA transcripts by competing with miRNAs^139^. Long noncoding RNAs (lncRNAs) can function as ceRNAs or “RNA sponges,” sequestering miRNAs and reducing their regulatory impact on target mRNAs^109^. For example, the lncRNA FAM225A promotes nasopharyngeal carcinoma (NPC) tumorigenesis and metastasis by acting as a ceRNA to sponge miR-590-3p and miR-1275, thereby upregulating ITGB3^140^. Additionally, 3’ UTR shortening plays a significant role in altering ceRNA expression. NUDT21, a key regulator of 3’ UTR alternative polyadenylation (APA), mediates these changes and can serve as a potential biomarker in tumorigenesis. When NUDT21 is knocked down, tumor suppressor genes such as PHF6 and LARP1 are repressed in an miRNA-dependent manner^141^. Similar ceRNA mechanisms have been reported for other lncRNAs, including H19 and HULC, further highlighting the critical regulatory interactions between lncRNAs and small noncoding RNAs in cancer progression^109^.

### Regulatory interactions of lncRNAs with mRNAs, DNA, and RNPs in cancer

Sense mRNA and antisense lncRNA transcripts can hybridize to form RNA duplexes, modulating the expression of the sense mRNA. Antisense lncRNAs can function as oncogenes or tumor suppressors in various cancer types and are also implicated in drug resistance^142^. For example, the lncRNA STAU1 mediates mRNA decay by interacting with double-stranded RNA regions in the 3′UTRs of target mRNAs^143^. TINCR lncRNA binds to several mRNAs containing 25-nucleotide TINCR box motifs, while lncRNA-p21 interacts with the JUNB and CTNNB1 mRNAs to selectively impair their translation^109^. LINC01089 promotes epithelial–mesenchymal transition (EMT), migration, invasion, and metastasis in HCC cells both in vitro and in vivo. LINC01089 knockdown increases the DIAPH3 protein level by affecting exon 3 of DIAPH3, which suppresses the ERK/Elk1/Snail axis and inhibits EMT in HCC cells. Overexpressed LINC01089 interacts with hnRNPM, inducing hnRNPM-mediated skipping of DIAPH3 exon 3. This exon contains a crucial m6A modification site recognized by IGF2BP3, which enhances DIAPH3 mRNA stability^144^. These findings illustrate the diverse regulatory interactions of lncRNAs with mRNA, DNA, and RNP complexes, highlighting their significant roles in cancer progression and potential therapeutic targets.

### Regulatory interactions between circRNAs and miRNAs in cancer

Circular RNAs (circRNAs) play significant roles in cancer by interacting with miRNAs to regulate gene expression. These compounds can promote the activity and expression of HIF-1α by sponging tumor suppressor miRNAs. Specifically, circRNF20, circ-03955, and circ-MAT2B sponge miR-487a, miR-3662, and miR-515-5p, respectively, to stabilize and prevent degradation of the HIF-1α transcript^145^. In pancreatic ductal adenocarcinoma (PDAC), circNEIL3 regulates the expression of ADAR1 by sponging miR-432-5p. The upregulation of ADAR1 leads to RNA editing of GLI1, which affects cell cycle progression and promotes epithelial–mesenchymal transition (EMT) in PDAC cells^146^. These interactions highlight the crucial role of circRNAs in modulating miRNA activity and influencing cancer progression.

## RNA-targeted and RNA-based therapeutics in cancer

### Therapeutics for targeting pre-mRNA-modifying enzymes with small molecules

Targeting enzymes or factors responsible for aberrant RNA modification has emerged as a promising therapeutic strategy for cancer treatment. Although no compounds specifically modulating pre-mRNA splicing have yet been approved for cancer treatment, several inhibitors targeting splicing factors, such as SF3B, SRPK, CLK, CDK, PRMT1, and PRMT5, are currently in clinical trials^147^. E7107 was the first splicing modulator to enter phase I clinical trials in patients with locally advanced or metastatic solid tumors^148^. Subsequently, H3B-8800, a promising orally available small molecule targeting SF3B1, entered phase I trials for treating hematological malignancies. These early efforts underscore the potential of targeting splicing modulation in clinical settings^149^.

Recent studies have also highlighted the potential of small-molecule inhibitors as a source of tumor antigens, which can be leveraged in immunotherapy^150,151^. For instance, triple-negative breast cancer (TNBC) cells produce many intron-retained double-stranded RNAs when treated with H3B-8800. These novel antigens activate the antiviral immune response and induce apoptosis^150^. Additionally, targeting RNA splicing with the sulfonamide derivative indisulam (E7070) degrades RBM39 in a dose-dependent manner, creating new antigens in cancer cells, stimulating an antitumor immune response, and enhancing the efficacy of immune checkpoint inhibitors^151^. Moreover, treatment with STM2457 has been shown to reduce acute myeloid leukemia (AML) growth and increase differentiation and apoptosis, demonstrating the broader therapeutic potential of targeting RNA modification enzymes in cancer therapy^152^.

### MicroRNA therapeutics in cancer

The use of microRNA (miRNA) therapeutics in cancer treatment began with the first clinical trial of small interfering RNA (siRNA) therapeutics in 2004, a mere six years after the discovery of RNA interference (RNAi). Clinical trials have demonstrated that siRNA therapeutics are generally well tolerated by patients^73^. The first miRNA therapeutic trial commenced in 2013, followed by the second in early 2015. Despite the similarities between siRNAs and miRNAs, progress in miRNA therapeutics has been relatively slow due to uncertainties in their mechanism of action and specificity. Numerous tumor suppressor miRNAs have been identified for their ability to downregulate oncogenes. These include miRNA-34, miRNA-16, miRNA-7, miRNA-126, miRNA-143/145, miRNA-200, miRNA-355, and members of the let-7 family^73^. Among these miRNAs, miRNA-34 is particularly well characterized as a natural regulator of tumor suppression and is downregulated in many cancers^153^. MRX34, a first-in-class cancer therapy and the first synthetic miRNA to enter clinical trials, was designed to deliver an miRNA-34 mimic using a liposomal formulation^154^. Another miRNA therapeutic that has reached the clinical trial stage is TargomiRs, an miRNA-16 mimic that is indicated for malignant pleural mesothelioma^155^. miRNA-16 is a tumor suppressor, and its restoration leads to the inhibition of tumor-promoting gene transcription^156^. In preclinical studies, combination therapy with miRNA-22 and lenvatinib has shown promising results. Compared to the FDA-approved monotherapy of lenvatinib, the combination therapy produced better survival outcomes without noticeable toxicity in a mouse model of HCC. This highlights the potential of miRNA-based therapies to enhance the efficacy of existing cancer treatments^157^.

### Antisense oligonucleotide therapeutics in cancer

Antisense oligonucleotide (ASO) therapeutics targeting specific mRNA sequences to modulate gene expression have shown significant promise in cancer treatment. One of the best-studied A-to-I RNA editing targets in cancer is antizyme inhibitor 1 (AZIN1). The RNA secondary structure of AZIN1 includes an editing site complementary sequence (ECS) at the 3’ end of exon 12. ASOs designed to target the editing region of AZIN1 have been shown to cause substantial exon 11 skipping, while ECS-targeting ASOs effectively abolish AZIN1 editing without affecting splicing or translation^158^.

Oblimersen (G3139) is a phosphorothioate 18-mer ASO designed to target the first six codons of the BCL2 gene. In a phase 1 study involving acute myeloid leukemia (AML) patients treated with G3139, no antisense-related toxicity was reported, and BCL2 downregulation was observed in patients who achieved complete remission^159^. ISIS3521, another antisense phosphorothioate oligonucleotide, targets protein kinase C alpha. Its efficacy and safety were tested in patients with relapsed low-grade non-Hodgkin’s lymphoma (NHL). Additionally, a phase II study was conducted to evaluate the clinical activity of ISIS3521 in patients with metastatic colorectal cancer^160^. Trabedersen (AP 12009), an inhibitor of TGF-beta2, underwent a phase IIb study to assess its efficacy and safety when administered intratumorally in patients with recurrent or refractory high-grade glioma^161^. These examples highlight the potential of ASO therapeutics for targeting specific genetic and epigenetic alterations in cancer.

### ASO therapeutics targeting lncRNAs in cancer

Antisense oligonucleotide (ASO) therapeutics targeting long noncoding RNAs (lncRNAs) offer promising strategies for cancer treatment by inducing the degradation or destabilization of lncRNAs. These methods include RNA interference (RNAi)-mediated gene silencing and ASO applications, both of which have shown significant therapeutic potential due to their high specificity, rational design, uniform chemistry, and simplified development cycle^162,163^. One notable example is Malat1, a lncRNA that has emerged as a compelling therapeutic target. ASOs targeting Malat1 have demonstrated potential for inhibiting breast cancer progression^164^ Similarly, AC104041.1, an oncogenic lncRNA, was targeted using LNA-modified ASOs designed to target two splice variants of AC104041.1, which exhibited potent antitumor activity in head and neck squamous carcinoma (HNSCC)^165^. Given their miRNA binding capacity, lncRNAs can be targeted to modulate miRNAs involved in HCC. For example, TRERNA1 is implicated in HCC progression through the regulation of miR-22-3p. Moreover, the upregulation of TRERNA1 is correlated with reduced responsiveness to sorafenib, indicating its potential as a therapeutic target in HCC^166^. The simultaneous targeting of these miRNAs using an artificial lncRNA expressed by an adenoviral vector (Ad5-AlncRNA) has been shown to inhibit proliferation, induce apoptosis in sorafenib-resistant cells, and enhance the effects of sorafenib both in vitro and in vivo^167^. Additionally, dominant-negative forms of endogenous lncRNAs can prevent their association with other binding partners involved in tumorigenesis. For example, the lncRNA HOTAIR recruits EZH2 to EMT-promoting sites to bind and repress SNAIL. Identifying the SNAIL-binding domain through bioinformatic fragmentation of HOTAIR led to the development of a therapeutic RNA molecule, HOTAIR-sbid (a HOTAIR deletion mutant). This dominant-negative version of HOTAIR contains the SNAIL-binding domain but lacks the EZH2-binding capacity, thereby inhibiting the tumor-promoting functions of endogenous HOTAIR^168^. These therapeutic approaches highlight the potential of ASOs targeting lncRNAs in modulating cancer progression and resistance, providing new avenues for effective cancer treatments.

### Therapeutics for targeting specific splicing pre-mRNA variants with SSOs

Splice-switching antisense oligonucleotides (SSOs) are synthetic short-stranded RNAs designed to base pair with cis-acting elements of target pre-mRNAs. This interaction facilitates the conversion of splicing isoforms by blocking the binding of splicing factors (SFs) to pre-mRNAs^169–173^. The therapeutic potential of SSOs has been recognized with FDA approvals, such as eteplirsen for Duchenne muscular dystrophy and nusinersen for spinal muscular atrophy, both in 2016^174^.

In the context of cancer, SSOs targeting specific splicing variants have shown significant promise. For instance, an SSO targeting exon 2 of BCL-X pre-mRNA significantly elevates the BCL-XS/BCL-XL ratio, promoting apoptosis in glioma cells^37^. Moreover, ASOs targeting specific RNA isoforms are emerging as potent pharmacological agents. SSOs that promote the splicing of MDM2-ALT1 have been shown to induce p53 protein expression and apoptosis in p53 wild-type cells^175^. Additionally, the isoform PKM2, commonly expressed in various cancers, has been targeted by SSOs to interfere with its expression, thereby promoting apoptosis in glioblastoma cell lines^176^. These advancements underscore the potential of SSOs in modulating pre-mRNA splicing to produce therapeutic effects, particularly in cancer treatment, by specifically altering the expression of splicing variants associated with disease progression and resistance.

## Conclusion

In this review, we explored the mechanisms underlying various RNA modifications and their roles in cancer development, highlighting the potential of RNA modifications in cancer therapeutics. Despite significant advancements in this field, several challenges and unanswered questions persist. There are numerous obstacles to the translation of RNA modification-based approaches into clinical practice. Identifying reliable biomarkers and developing targeted therapies necessitate large-scale validation studies, integration with other molecular profiling data, and a thorough understanding of the heterogeneity and dynamics of RNA modifications in cancer. Additionally, ensuring the efficient delivery, stability, and minimal toxicity of RNA modification-modulating agents to tumor tissues while reducing off-target effects remains a critical hurdle in therapeutic development.

Continued research into the mechanisms, functional consequences, and clinical implications of RNA modifications in cancer will deepen our understanding of this disease and may pave the way for innovative diagnostic and therapeutic strategies. By elucidating the functional consequences of RNA dysregulation in cancer, we can uncover new avenues for therapeutic intervention, offering hope for more effective cancer treatments in the future.
